# Supplementary material for: Oxygen evolution from extremophilic cyanobacteria confined in hard biocoatings
Source: Microbiol Spectr. 2023 Sep 25;11(5):e01870-23. doi: 10.1128/spectrum.01870-23 (PMC10580922; doi:10.1128/spectrum.01870-23)
Supplement: Supplemental figures — Fig. S1 to S15. [file spectrum.01870-23-s0001.docx]

Supplemental Material

Oxygen evolution from extremophilic cyanobacteria confined in hard biocoatings

Simone Krings^1^, Yuxiu Chen^2*^, Joseph L. Keddie^2^, Suzanne Hingley-Wilson^1^

1 Department of Microbial Sciences, School of Biosciences

2 School of Mathematics and Physics,

University of Surrey, Guildford, Surrey, GU2 7XH, United Kingdom

* Present address: School of Engineering, University of Newcastle, Newcastle upon Tyne, NE1 7RU, United Kingdom

For correspondence: [s.hingley-wilson@surrey.ac.uk](mailto:s.hingley-wilson@surrey.ac.uk), [j.keddie@surrey.ac.uk](mailto:j.keddie@surrey.ac.uk)

1. **Sample preparation for imaging by SEM/EDX and CLSM**

For SEM/EDX and CLSM, samples were prepared on 22 mm × 22 mm coverslips whose edges were redrawn with a 2 mm tip Liquid Blocker Pen (PAP Pen) (Agar Scientific, AGL4197S, Stansted, UK) on which 110 µL of the suspension were placed and film-formed. The dried biocoatings were rehydrated by adding 300 µL BG11(-SW). Samples were frozen at -80°C overnight and placed in a freeze-drier (Edwards Modulyo, Bristol, UK) at -40°C at 60-80 mbar for 24 hours and stored in desiccators containing silica gel until use.

Cross-sectioning for SEM/EDX was achieved by either cryo-fracturing or cryo‑sectioning. For cryo-fracturing, the glass substrates were first nicked using a diamond knife and samples were cryo-fractured after submersion in liquid nitrogen in order to preserve the microstructure of the biocoatings. To prepare for cryo-sectioning, freeze-dried biocoating samples were embedded in Scigen Tissue-Plus™ O.C.T. Compound (Fisher, 23-730-571, Loughborough, UK) and frozen at -80°C. Samples were then sectioned in 7 µm slices with a cryostat (Leica, CM3050 S, Milton Keynes, UK) and placed onto coverslips.

1. **SEM and EDX**


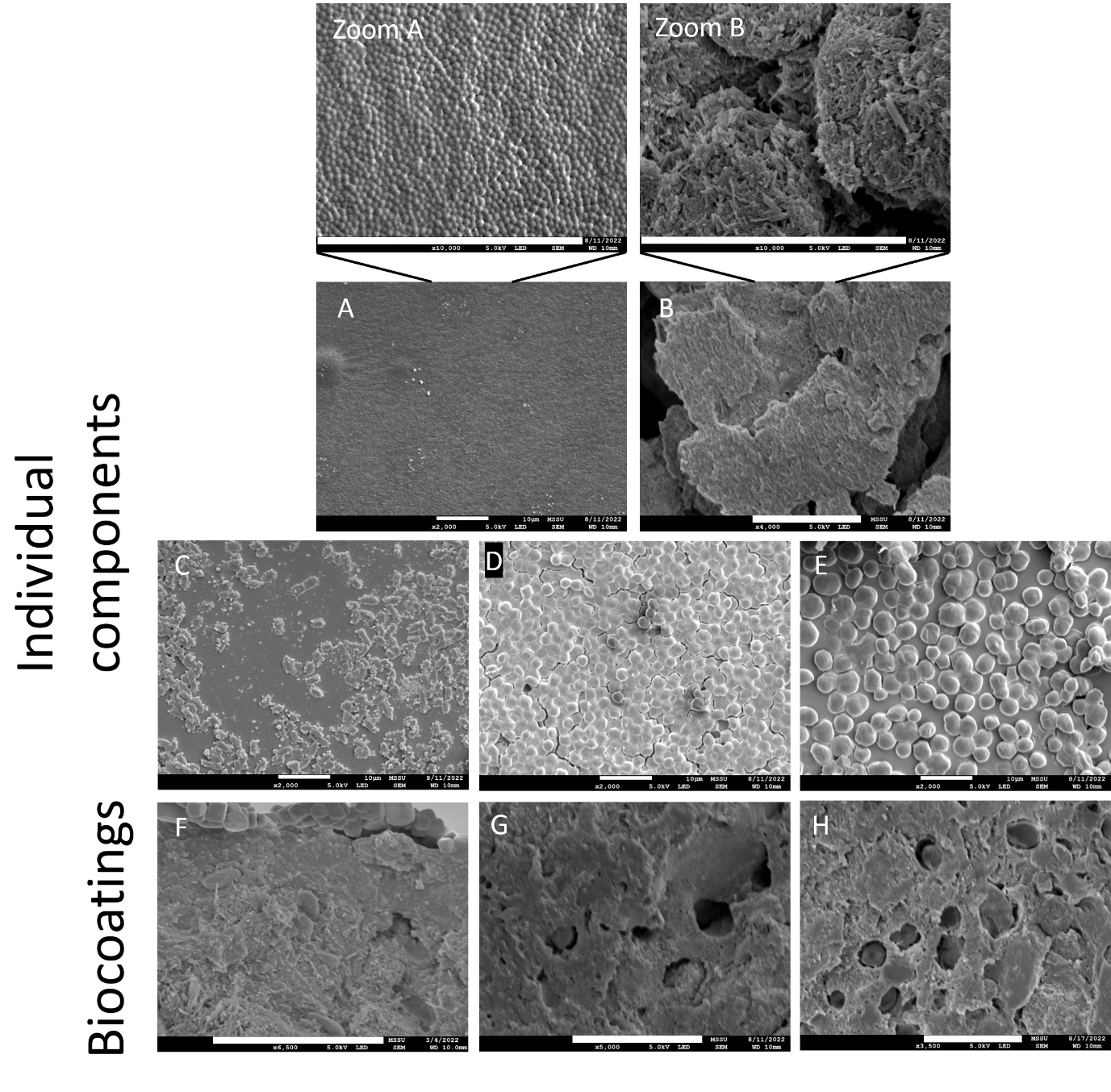


**Figure S1. Scanning electron microscopy images of biocoating components separately** (A-H) (controls) (A) Latex, (B) Halloysite, (C) *Synechococcus* sp. PCC 7002, (D) *Synechocystis* sp. PCC 6803, (E) *Chroococcidiopsis cubana* sp. PCC 7433 **and after film formation and rehydration** (F-G) (biocoatings) (F) *Synechococcus* sp. PCC 7002, (D) *Synechocystis* sp. PCC 6803, (E) *Chroococcidiopsis cubana* sp. PCC 7433.

Scale bar: 10 µm


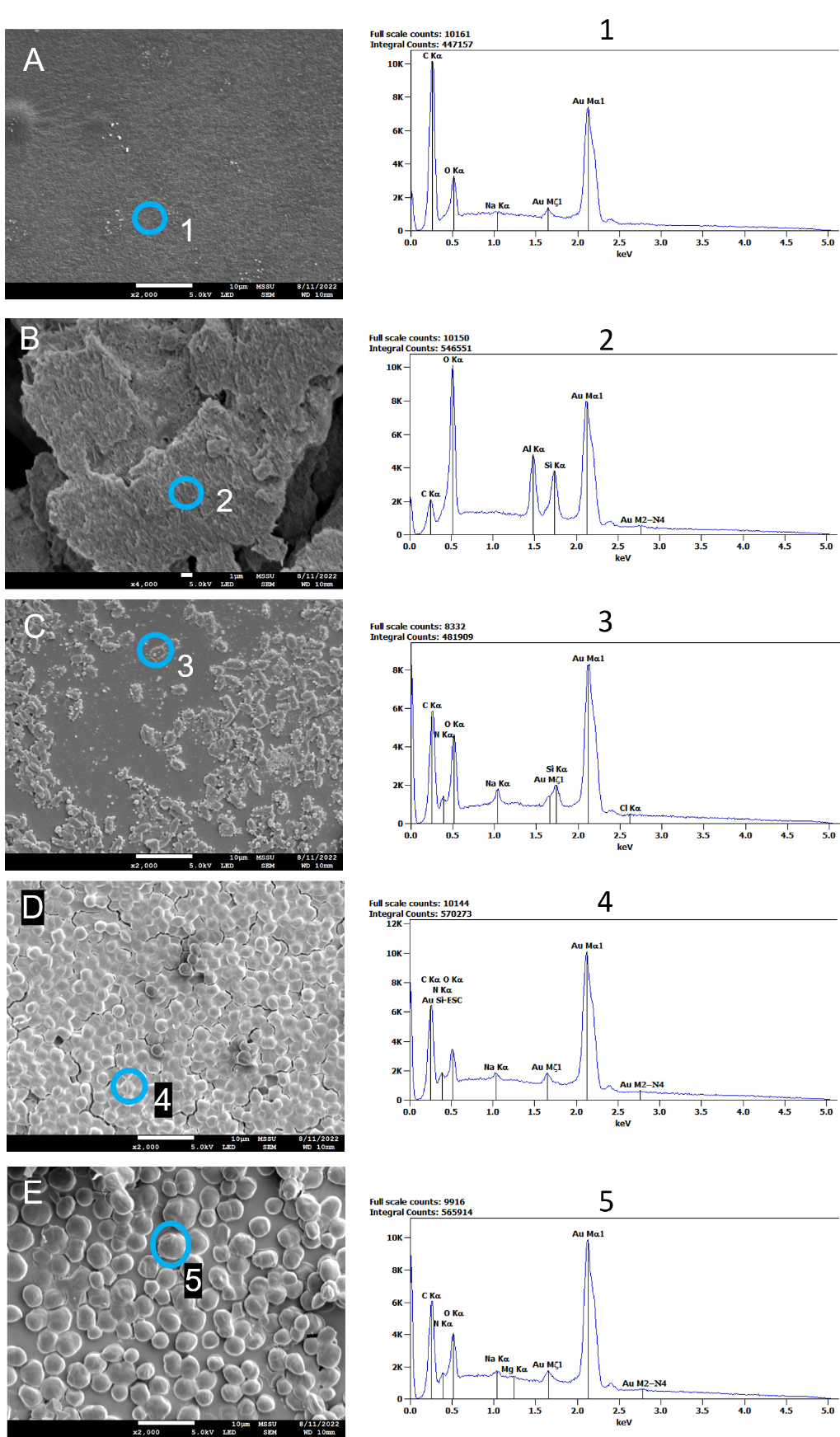


**Figure S2. Spectra from EDX analysis for the identified points from the individual components.**


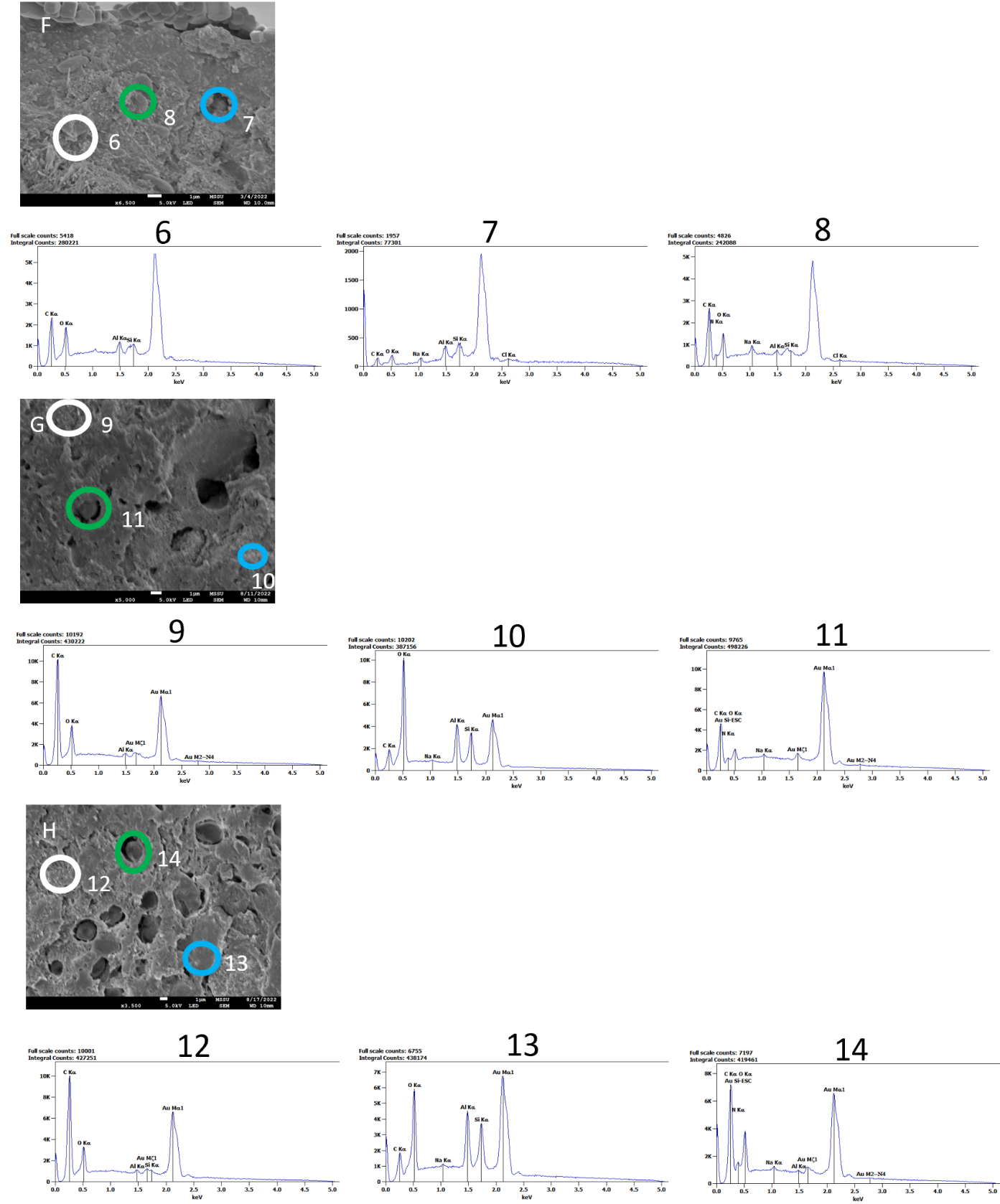


**Figure S3. Spectra from the EDX analysis for the presented points in biocoatings.**

1. **Cell viability**

In preliminary experiments, *Chroococcidiopsis* 7433 was dried on dried on BG11 agar for one month and the dried sheet could be transferred onto a fresh agar plate. After one week, these bacteria recovered their viability and were able to produce gases.


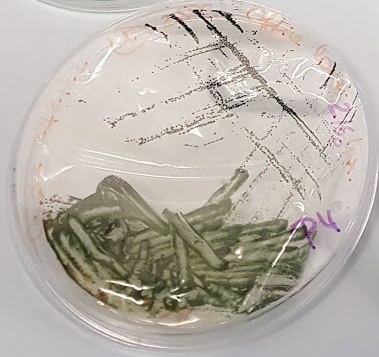

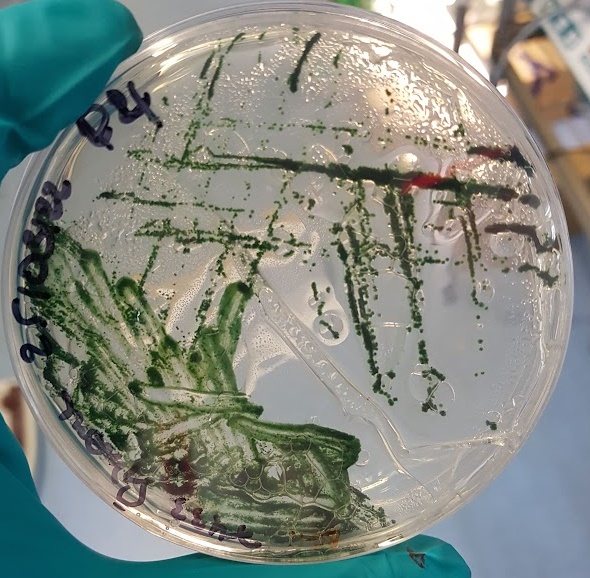


Dried for 1 month 1 week later on fresh BG11

**Figure S4. *Chroococcidiopsis* 7433 could be revived from dried colonies.** *Chroococcidiopsis* 7433 was dried on BG11 agar for one month, which resulted in dried colonies on a sheet of BG11 agar. This sheet of dried BG11 agar containing the dried colonies was transferred onto a fresh BG11 agar plate. After one week, the bacteria recovered their viability and produced gases.

Standard curves were established using the ten-fold diluted cyanobacteria or Adenosine 5’-triphosphate disodium salt hydrate (Sigma-Aldrich, A7699) over relative luminescence units (RLU). These values were normalised against the negative controls. The standard curves and their equations were then used to estimate the viability of the bacteria within the dried samples (including the biocoatings).


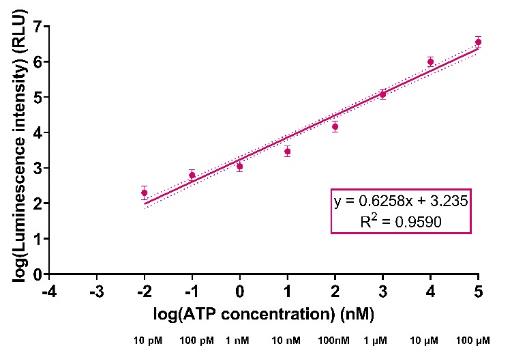


**Figure S5. Standard curve of Luminescence intensity in log_10_(RLU) over ATP concentration in log_10_(ATP) (nM) from the CellTiter-Glo® 3D viability assay.** A linear regression was fitted on the data (R^2^ = 0.9590) and the confidence intervals at 95 % are represented as dotted lines. The best-fit linear equation on the log-log axes was used subsequently to estimate the ATP concentrations from measured RLU values. (Mean ± SD, *n* = 3)


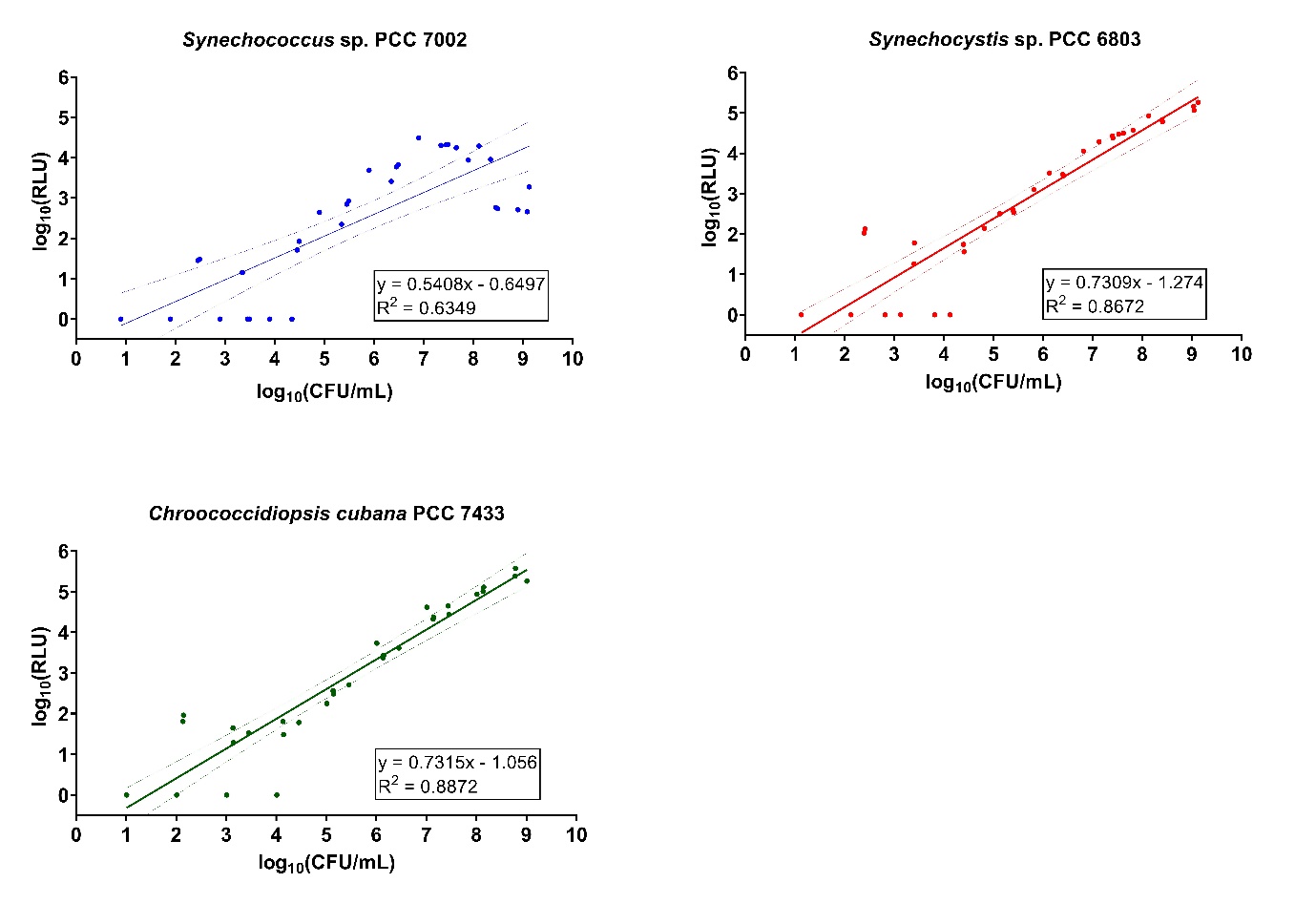


**Figure S6. Cyanobacterial concentration in log_10_(CFU/mL) over luminescence intensity in log_10_(RLU) for three bacterial species.** The liquid samples were diluted in ten-folds, measured using the CellTiter™ assay and normalised against the negative controls. The linear regression and the 95% Confidence intervals are represented. Although low RLU values for the lower CFU/mL could be detected occasionally for all three species, in the case of *Synechococcus* sp. PCC 7002 lower RLU values within the highly concentrated bacteria (> 8.5 log_10_ CFU/mL) could also be found. The assay is unreliable for this strain resulting in the lower R^2^ observed.


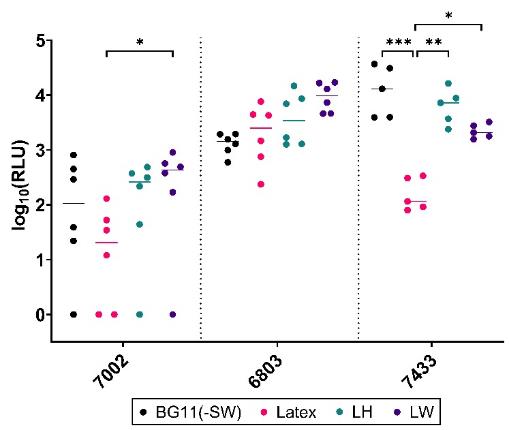


**Figure S7. Normalised luminescence intensity against negative controls of the 3 cyanobacterial strains that were dried in the different conditions.** All samples containing *Synechococcus* sp. PCC 7002 had the lowest RLU values, as well as *Chroococcidiopsis cubana* PCC 7433 in Latex, indicating low survival of the bacteria in these samples. For *Synechocystis* sp. PCC 6803, LW samples showed the highest survival which suggests that halloysite did not have a beneficial effect in these bacteria. *Chroococcidiopsis cubana* PCC 7433 had very high survival in BG11 (not encapsulated), followed by LH and LW. (Mean with biological replicates, *n* = 6 for 7002 and 6803, *n* = 5 for 7433, *: *p* ≤ 0.05, **: *p* ≤ 0.01, ***: *p* ≤ 0.001)

1. **Oxygen evolution from biocoatings**
   1. **Set-up with the oxygen sensors**


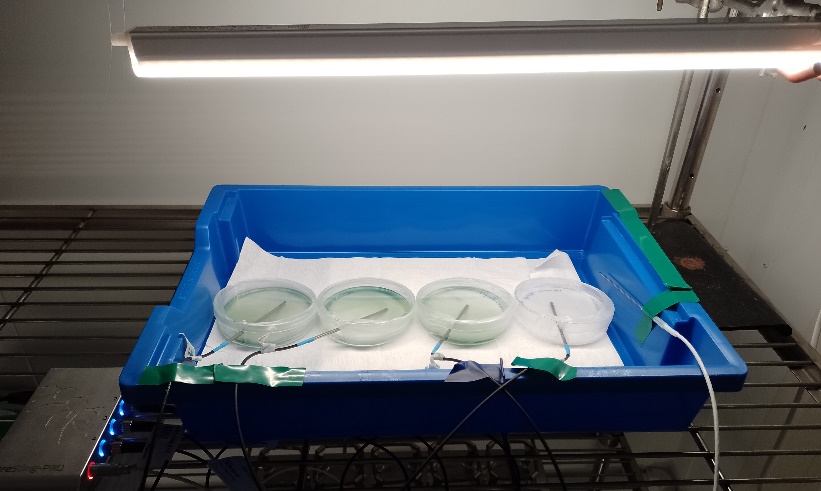


**Figure S8. Set-up of the oxygen evolution experiments with the oxygen fibre sensors. The lamp is positioned above four biocoatings in Petri dishes. The oxygen sensor probes are placed in the liquid above the biocoatings.**

Day 0


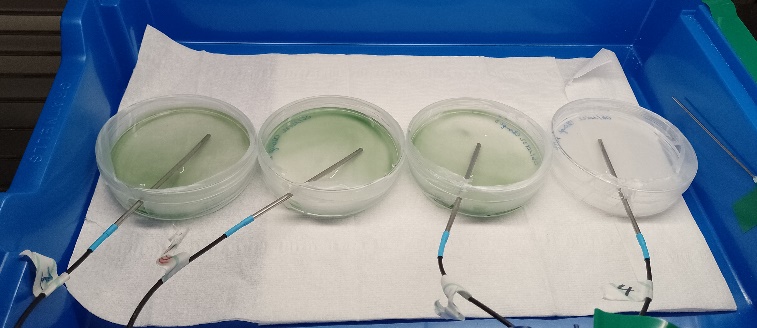


Day 12


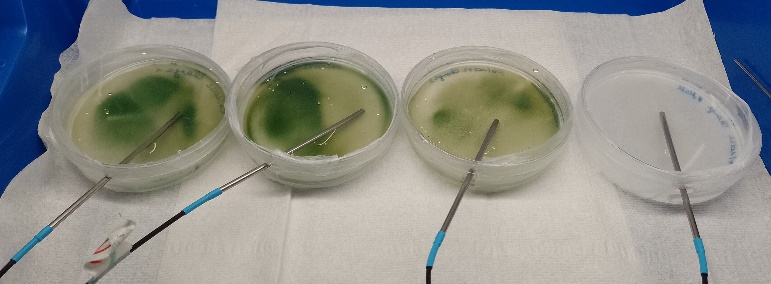


Day 27


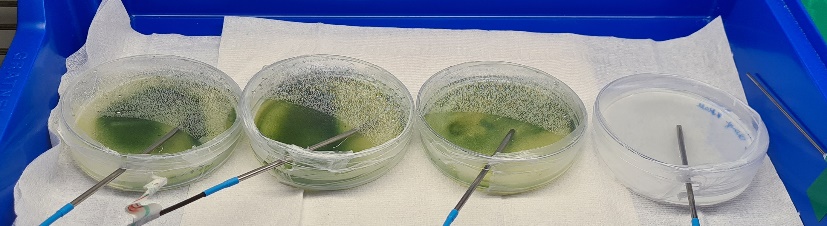


**Figure S9. Photographs of long-term experiment showing four biocoatings in Petri dishes with oxygen sensors inserted.**

- 1. **Dried vs. Liquid bacteria**
  - *Synechocystis* sp. PCC 6803


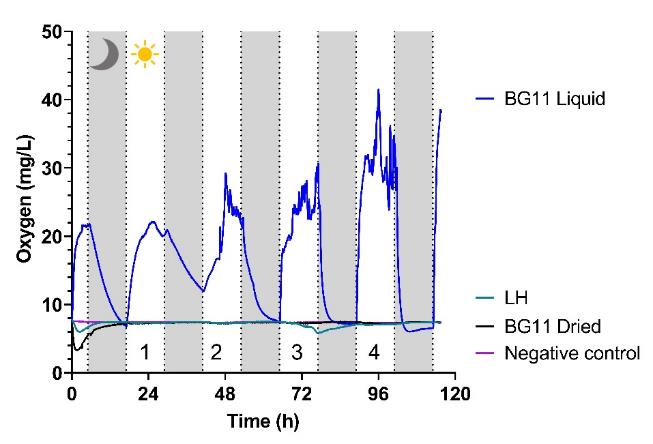


**Figure S10. No oxygen evolution could be observed from dried *Synechocystis* sp. PCC 6803.** A film-formed biocoating (LH) was compared to a dried culture (BG11 Dried), liquid culture (BG11 Liquid) and a negative control. The liquid culture evolved oxygen, while dried samples did not.

(*n* = 1, all samples contained 11.70 ± 0.08 log CFU/m^2^)

- *Chroococcidiopsis cubana* PCC 7433


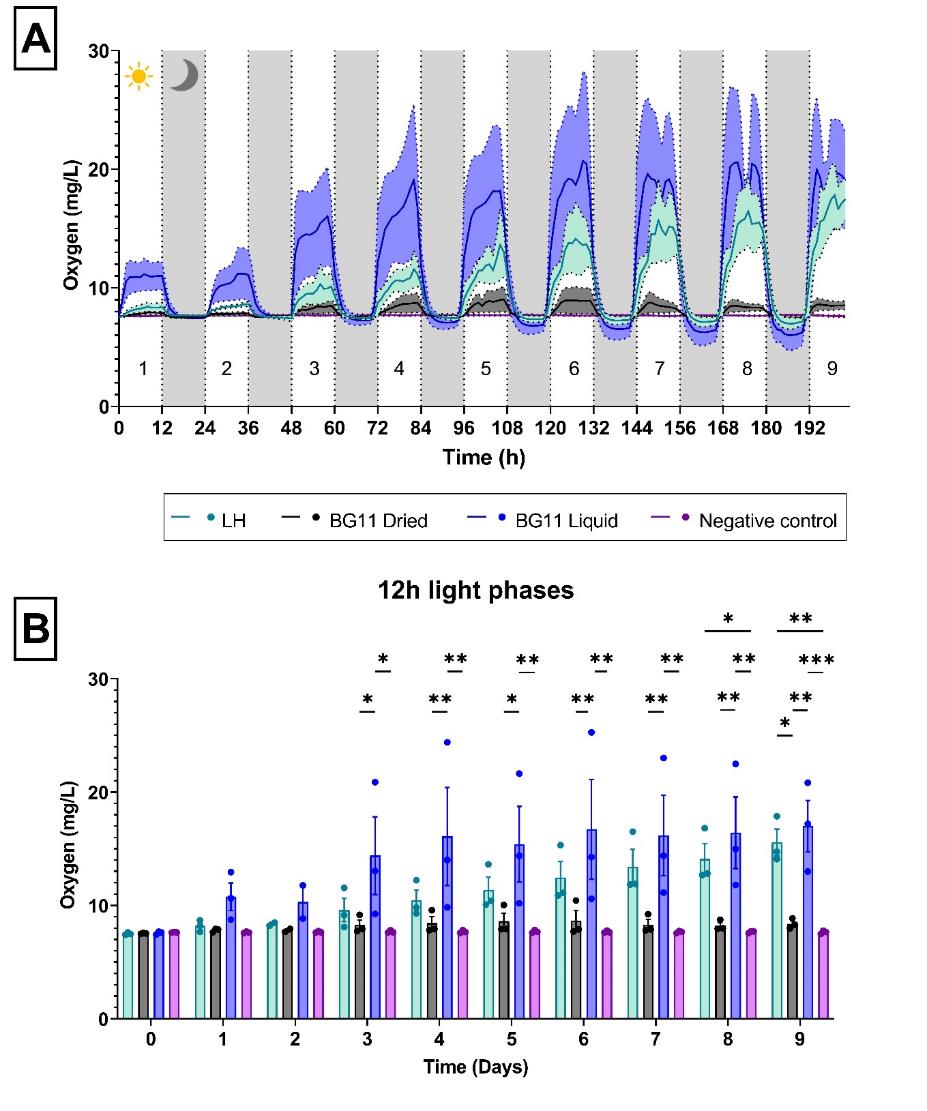


**Figure S11. Oxygen evolution was observed from liquid cultures and LH biocoatings.** The film-formed biocoatings (LH) were able to produce oxygen, although less than liquid cultures (BG11 Liquid), but more than dried cultures (BG11 Dried).

(*n* = 3, Mean ± SEM, *: *p* ≤ 0.05, **: *p* ≤ 0.01, ***: *p* ≤ 0.001, all samples contained 9.45 ± 0.74 log CFU/m^2^)

- 1. **Light intensity of *Chroococcidiopsis cubana* PCC 7433 in biocoatings**


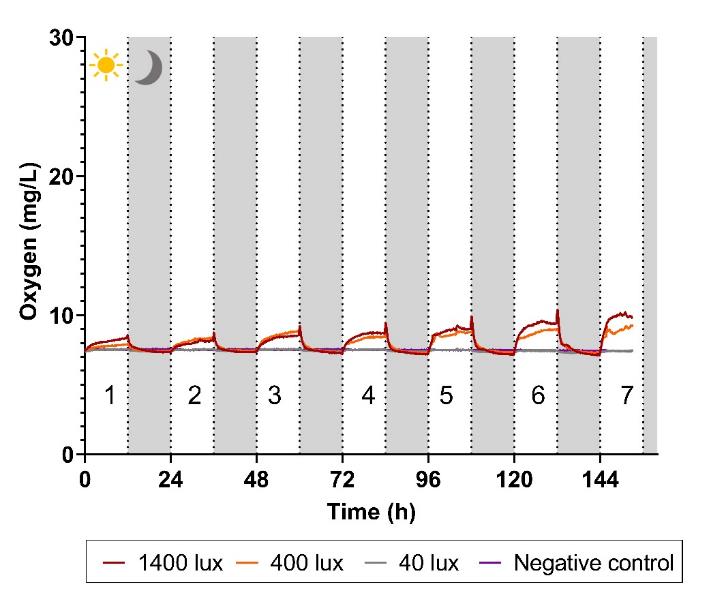


**Figure S12. The influence of light intensity on oxygen evolution of LH biocoatings containing *Chroococcidiopsis cubana* PCC 7433.** The film-formed biocoatings (LH) were exposed to 1400 lux, 400 lux and 40 lux. Oxygen evolution under 1400 lux and 4000 lux was similar whereas no oxygen production was observed under 40 lux. The biocoatings contained 9.95 log CFU/m^2^. (*n* = 1)

- 1. **Concentration of *Chroococcidiopsis cubana* PCC 7433 in biocoatings**


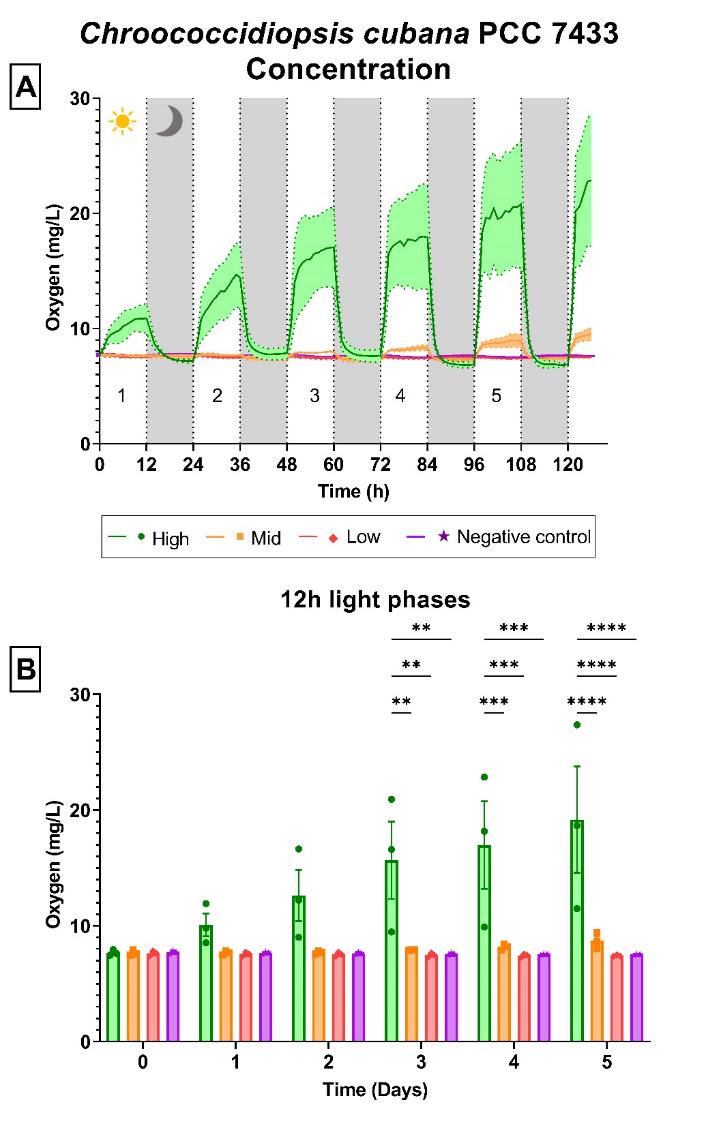


**Figure S13. High concentrations of *Chroococcidiopsis cubana* PCC 7433 in LH biocoatings resulted in higher levels of oxygen production.** The bacterial concentrations were: For High: 10.31 ± 0.16 log CFU/m^2^, Mid: 9.90 ± 0.02 log CFU/m^2^, Low: 8.95 ± 0.06 log CFU/m^2^. The high concentration was therefore chosen for the experiments described in the main article.

(*n* = 3, Mean ± SEM, **: *p* ≤ 0.01, ***: *p* ≤ 0.001, ****: *p* ≤ 0.0001)

1. **Calculation of the specific rate of oxygen production of *Chroococcidiopsis cubana* PCC 7433 in the species experiment**


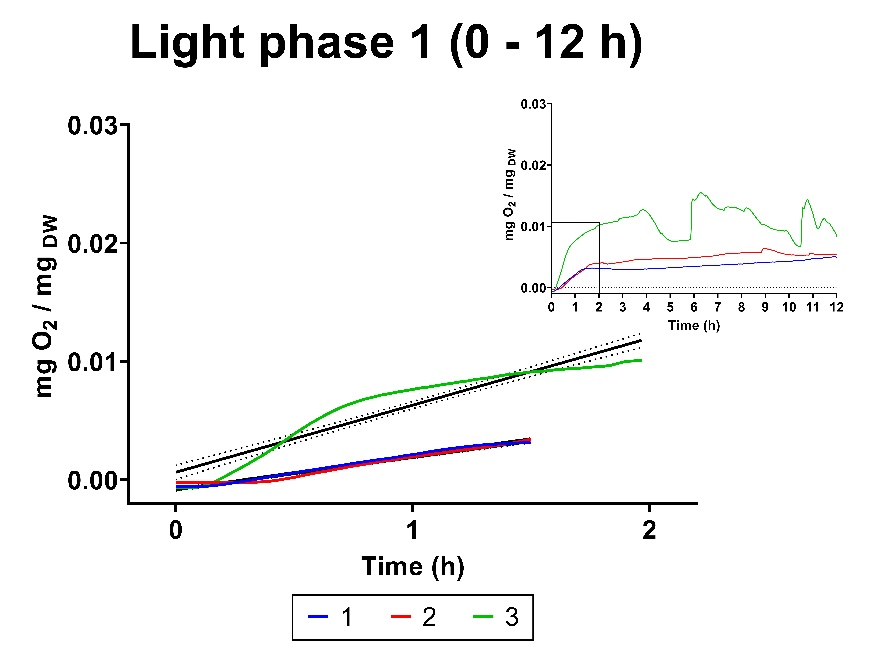

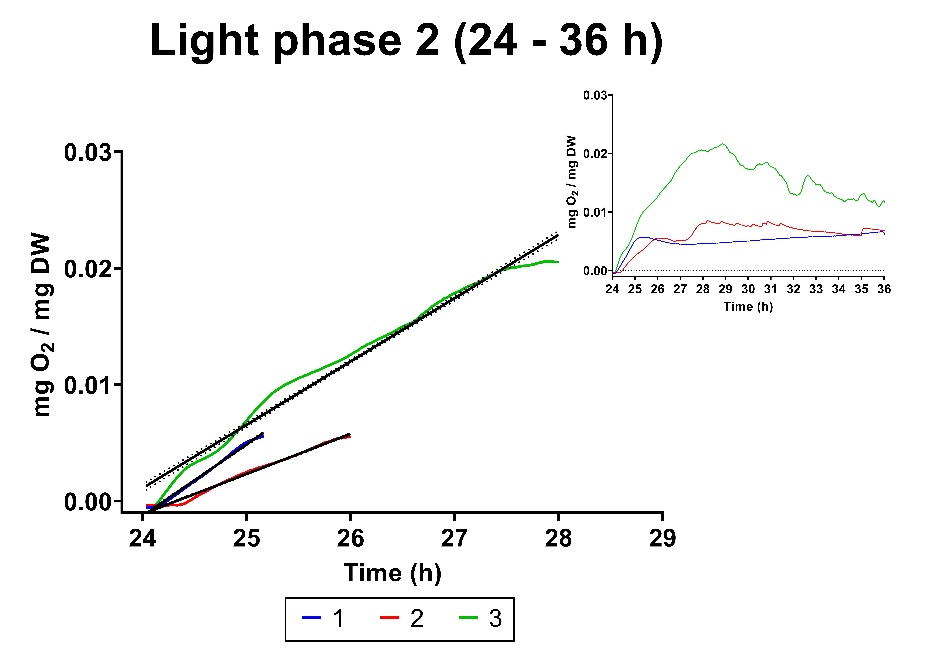


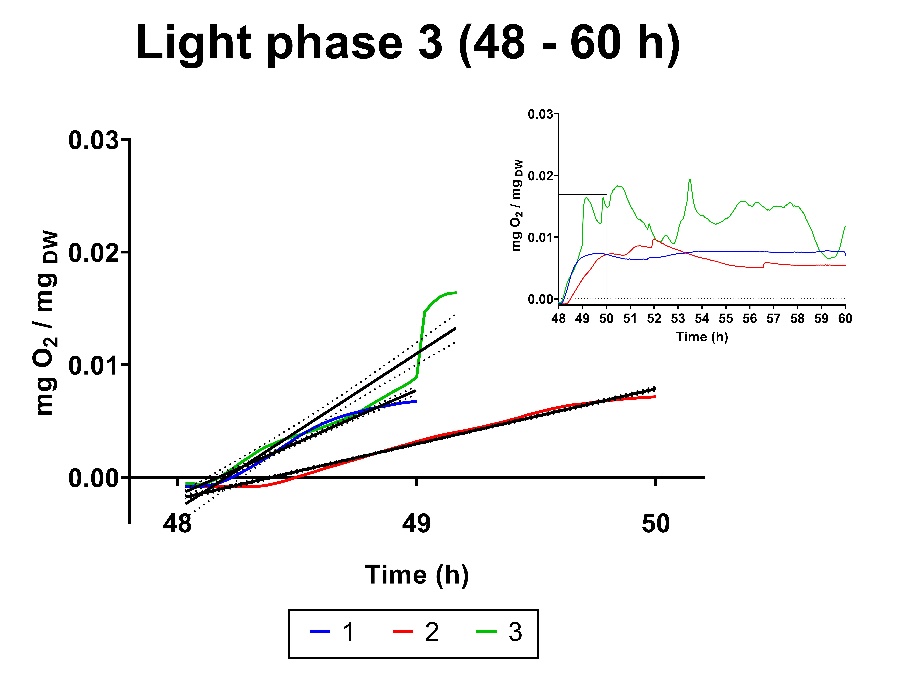


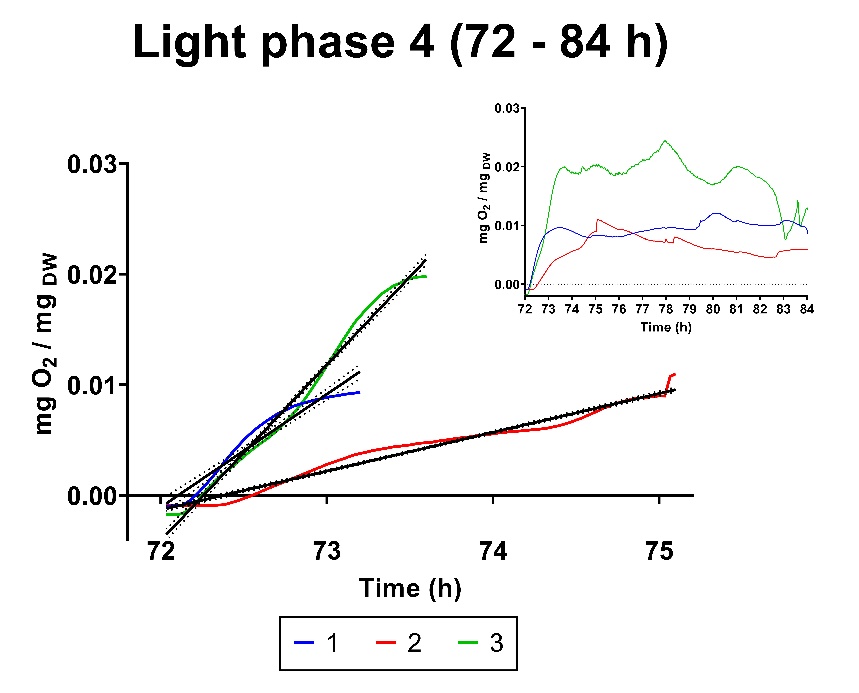


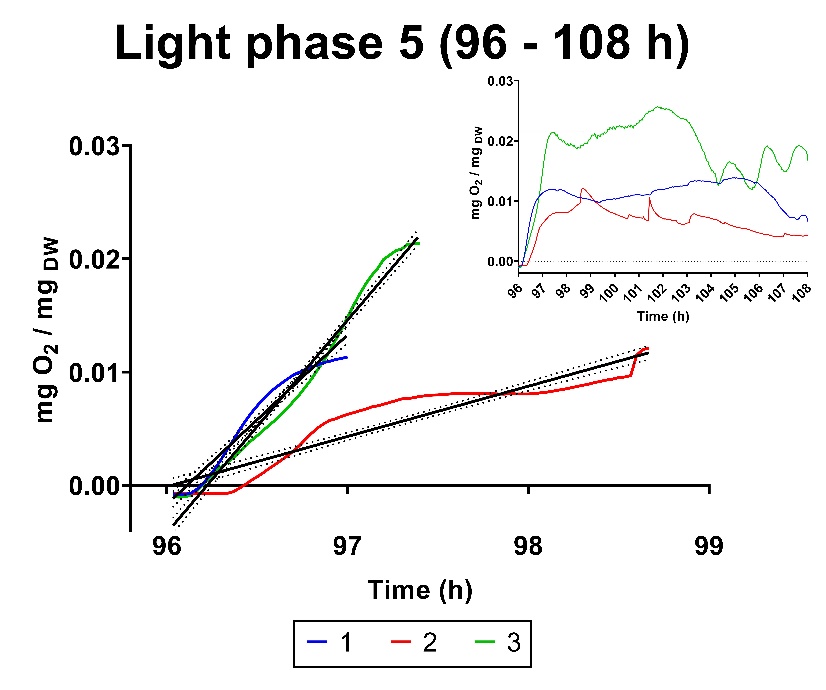


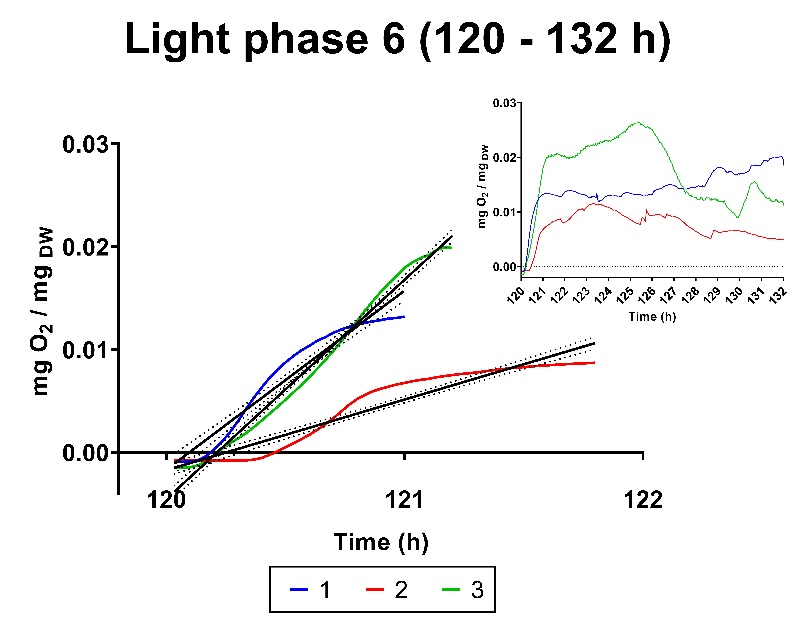


**Figure S14.** The specific rate of oxygen production by *Chroococcidiopsis cubana* PCC 7433 as measured from the linear gradients of plots of the mass of oxygen (in the medium volume of 20 mL) against time during the beginning of the light phase. The gradients are taken from the first hours where the data are linear. Analyses for six successive 12-hour light phases are presented. Replicates 1 and 3 were highly similar, while replicate 2 needed more time to reach the maximum rate of production.

1. **Flow cytometry gating strategies**

As described for confocal laser scanning microscopy, the viable bacteria contain chlorophyll *a* fluorescing red (YL3 channel: 561 nm excitation laser and 695/40 filters), which is degraded when the bacteria died, leaving only green fluorescence (BL2 channel: 488 nm excitation laser and 590/40 filters). The forward and side light scatters (FSC and SSC, respectively), as well as the channels BL2, YL3 and YL4 were used for the gating.

The voltages were set to FSC: 200 V, SSC: 320 V, BL2: 400 V, YL3: 280 V and YL4: 360 V. The Acquisition volume was 50 µL and the flow rate at 12.5 µL/min to record a total of 10 000 events. Acquired data were analysed using FlowJo v.10.8.1 software. First, gating in FSC-A (FSC-Area) vs. SSC-A (SSC-Area) was used to separate Cells and counting beads. The resulting counting beads population was then regated on FSC-A vs. YL4-A, resulting in the final ‘beads’ populations. To eliminate cell debris and ensure the analysis of single cells only, the ‘Cells’ population was regated on FSC-A vs. FSC-H (height), resulting in the final ‘Single cells’ population. This resulting population was then gated on BL2-A vs. YL3-A (logarithmic scale), allowing to differentiate between live (YL3+, BL2-) and dead (YL3-, BL2+) populations. To calculate the cell concentration (live cells/mL), the counts of the bead events and live cell events were then used in the formula [1, 2]. As the rehydration liquid was concentrated from 20 mL into 1 mL (prior to the flow cytometry), this concentration was divided by 20 to obtain the concentration (in cells/mL) of the original rehydration liquid.

$$Absolute count \left( \frac{cells}{mL} \right)=\frac{\frac{\left( Cell count \left( cells \right)\times Beads volume \left( mL \right) \right)}{\left( Beads count \left( beads \right)\times Cell volume \left( mL \right) \right)}}{20}$$

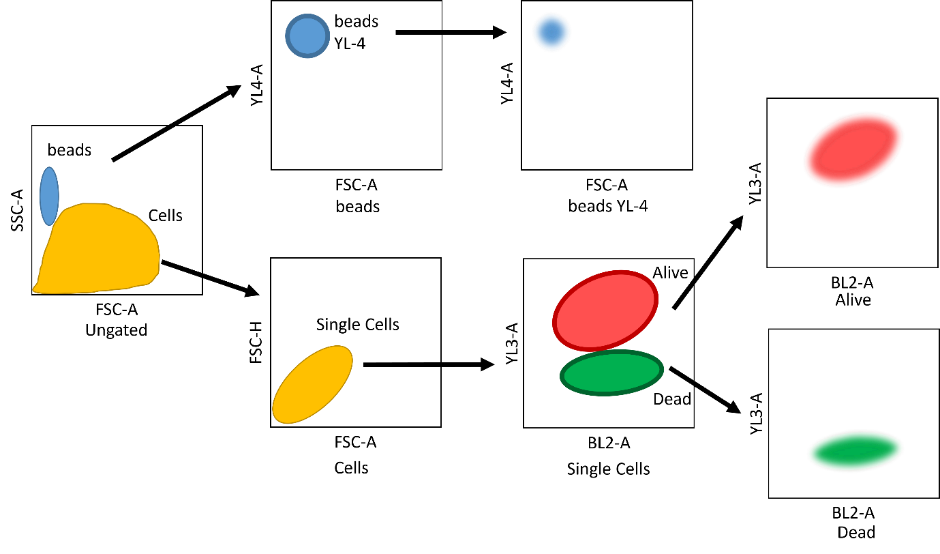


**Figure S15.** **Gating strategies for flow cytometry.** The ungated population was gated into beads and cells (on FSC-A vs. SSC-A). The beads population was gated into beads YL-4 (on FSC-A vs. YL4-A), resulting in the final beads population whose absolute counts were used for calculations. The cells population was gated into single cells (diagonally on FSC-A vs. FSC-H). This population was then gated into live and dead populations (on YL3-A vs. BL2-A), where live cells fluoresce more red (due to chl *a*), compared to dead cells. The resulting counts were used for the calculations.

- Live and Killed samples (10^-1^ dilution)
  - 7002 – page 16
  - 6803 – page 17
  - 7433 – page 18
- Escaped bacteria (Species oxygen experiment)
  - 7002 – page 19
  - 6803 – page 20
  - 7433 – pages 21
- Long-term oxygen experiment with *Chroococcidiopsis cubana* PCC 7433
  - Live and killed samples used for set-up – pages 22 & 23
  - Escaped bacteria D12 and D27 – pages 24 & 25

*Synechococcus* sp. PCC 7002: Live and Dead gating on live and killed samples


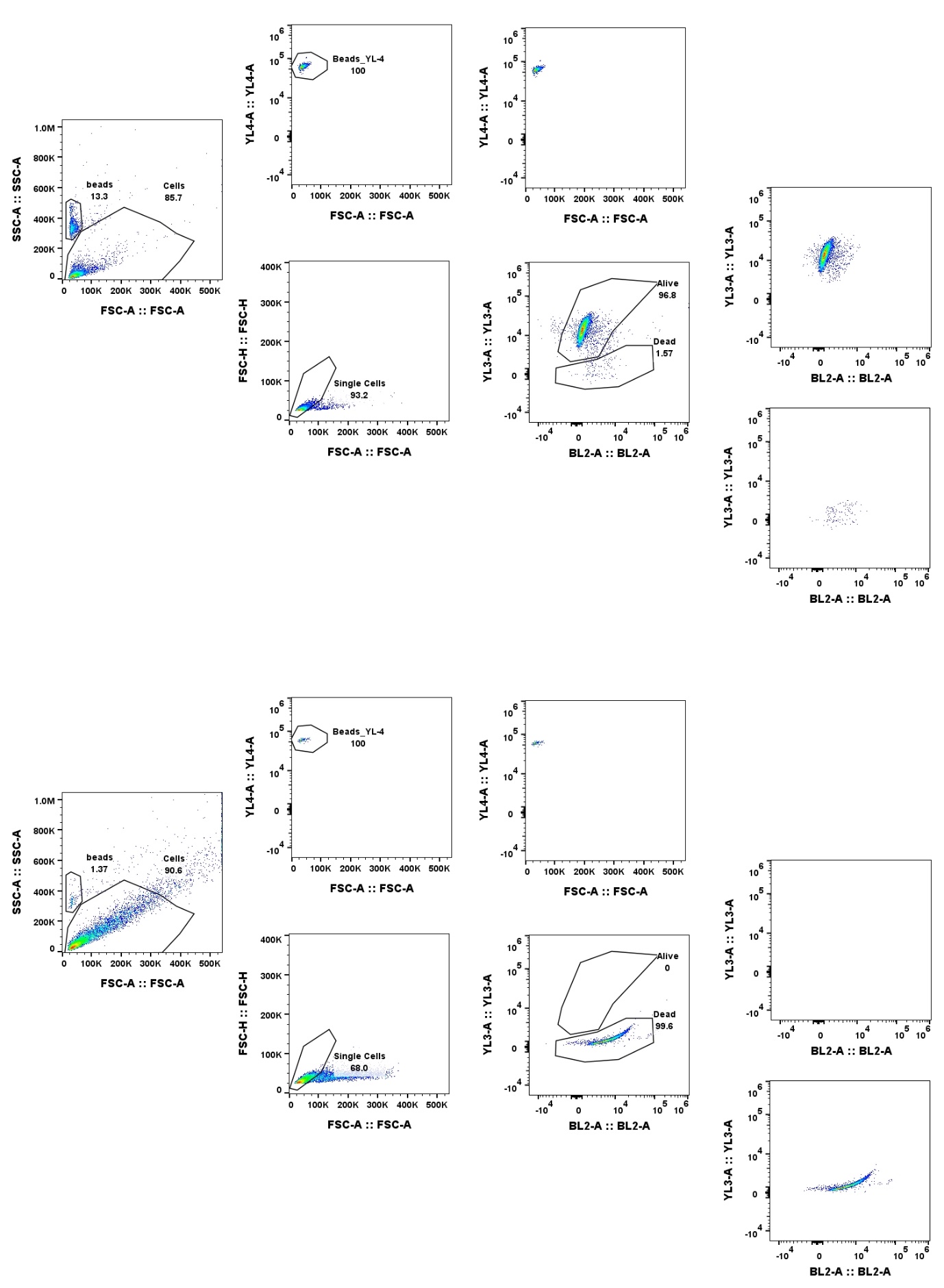


*Synechocystis* sp. PCC 6803: Live and Dead gating on live and killed samples


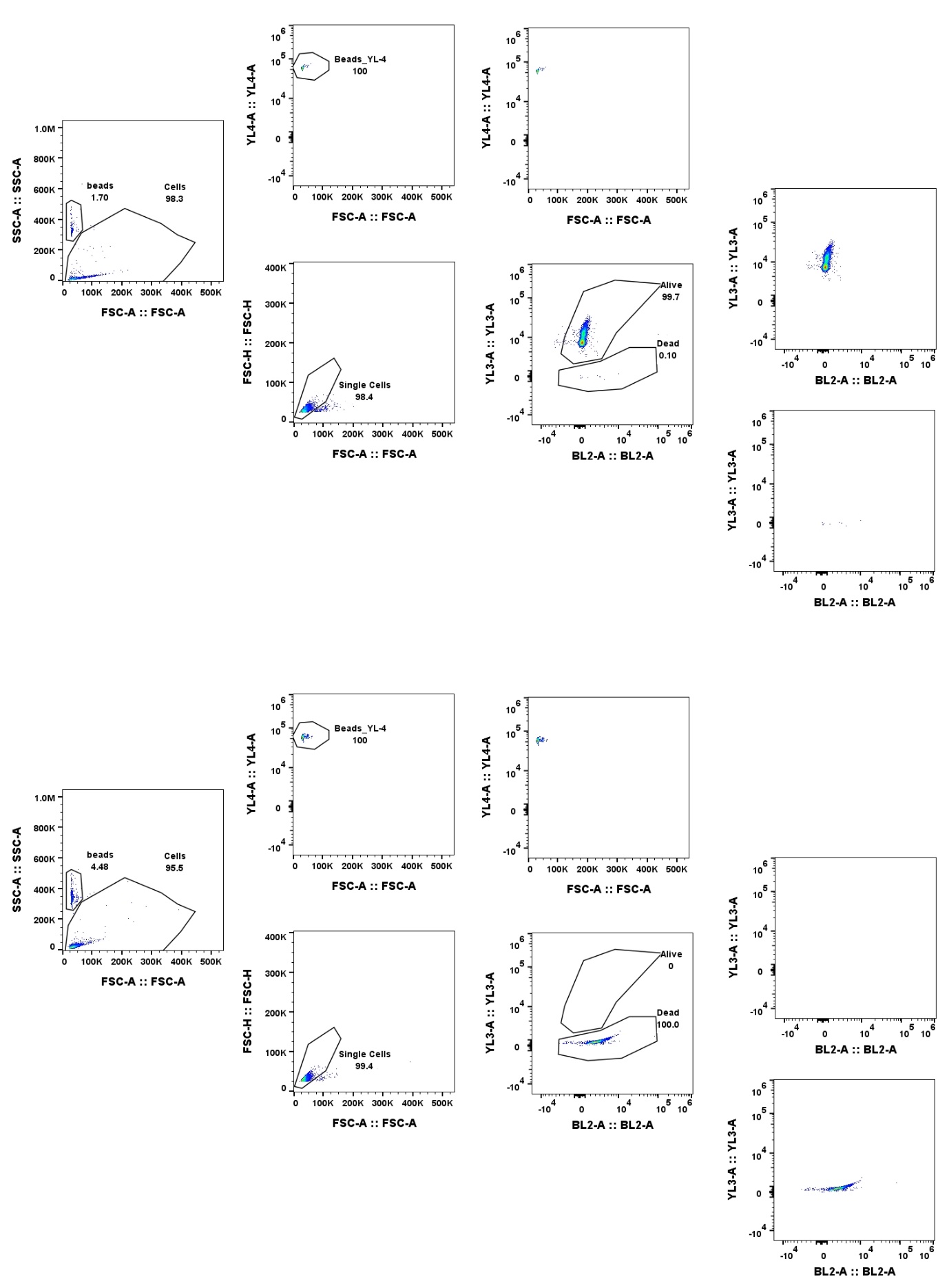


*Chroococcidiopsis cubana* PCC 7433: Live and Dead gating on live and killed samples


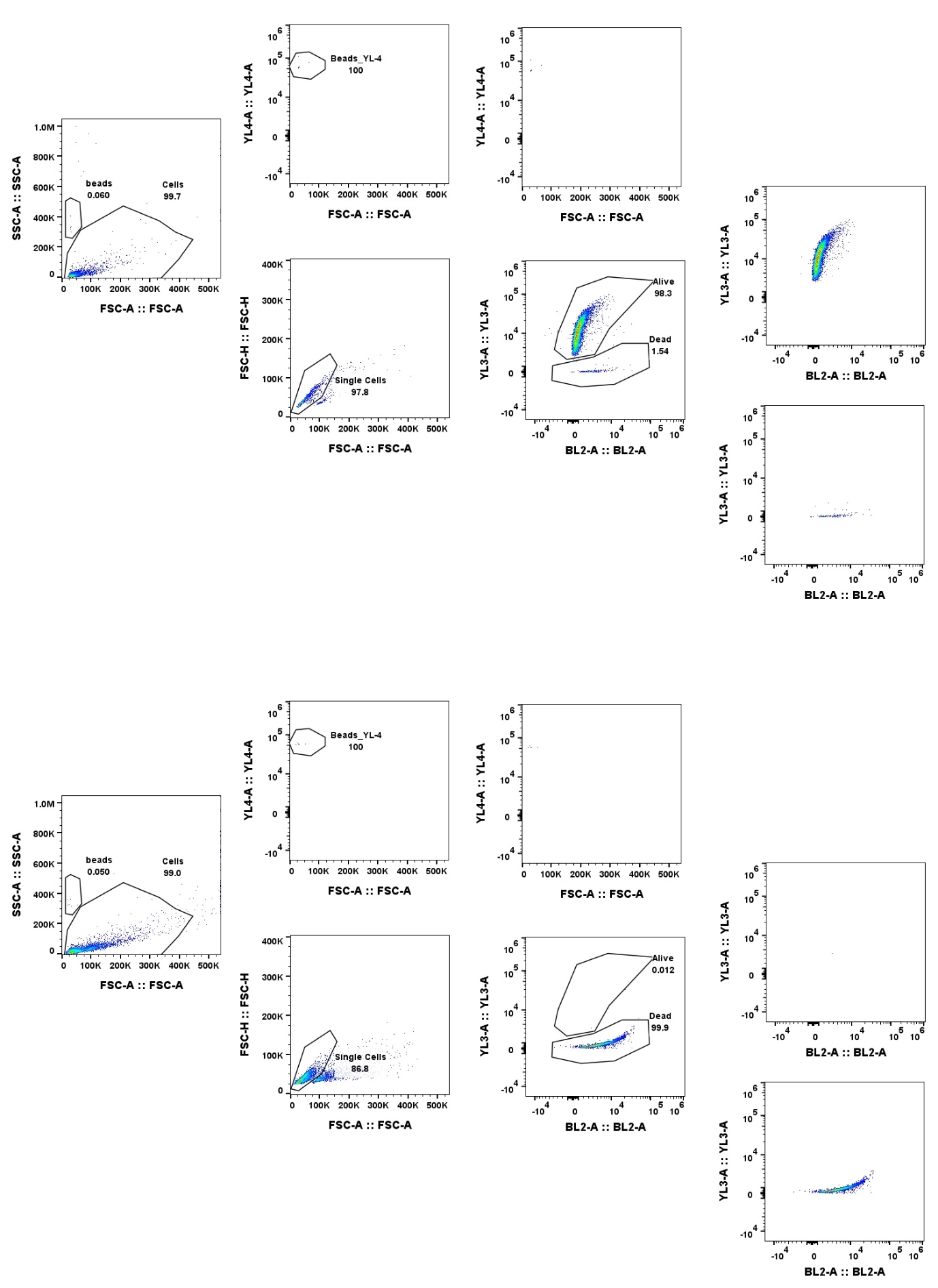


*Synechococcus* sp. PCC 7002: Escaped bacteria
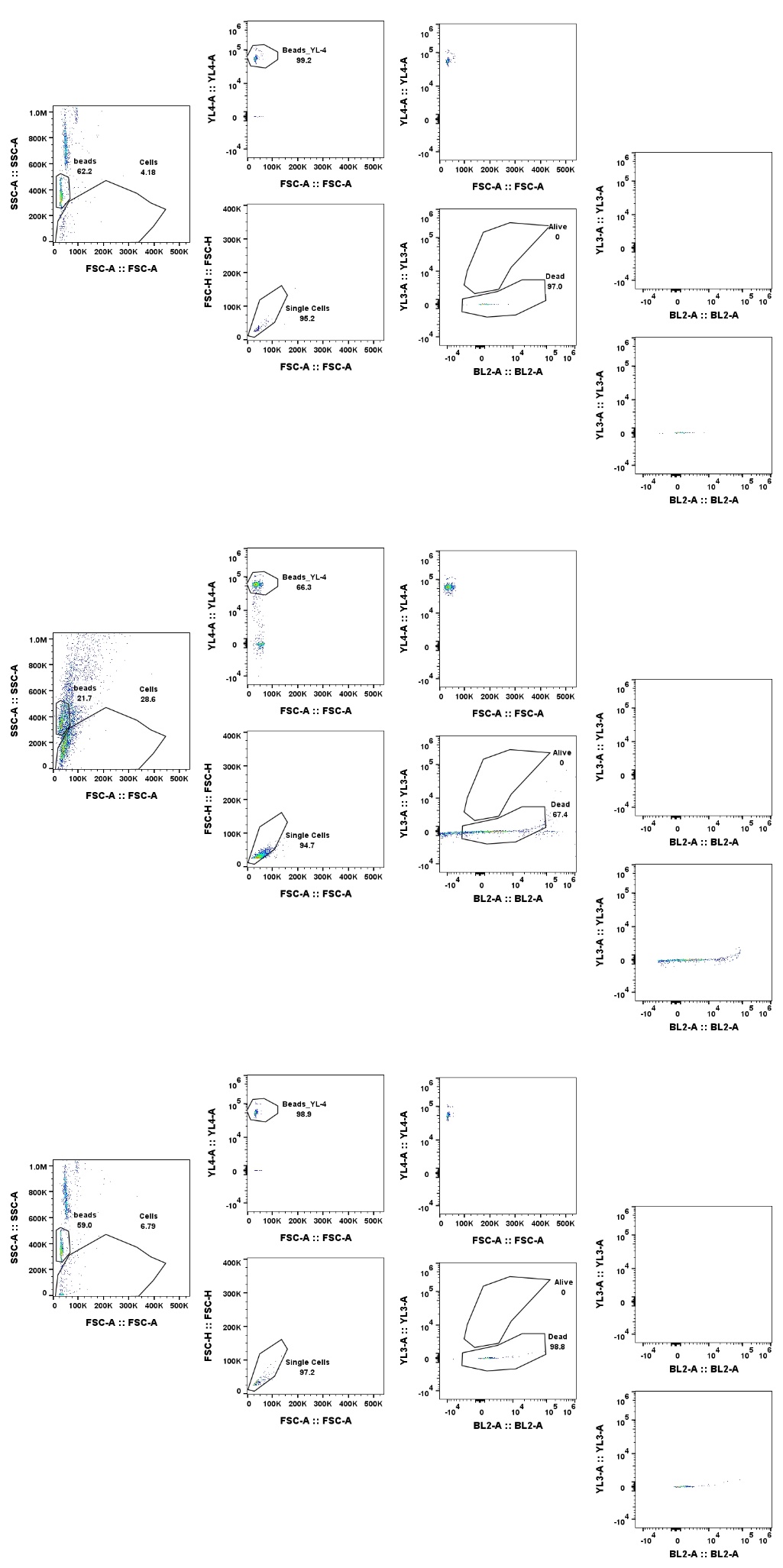


*Synechocystis* sp. PCC 6803: Escaped bacteria
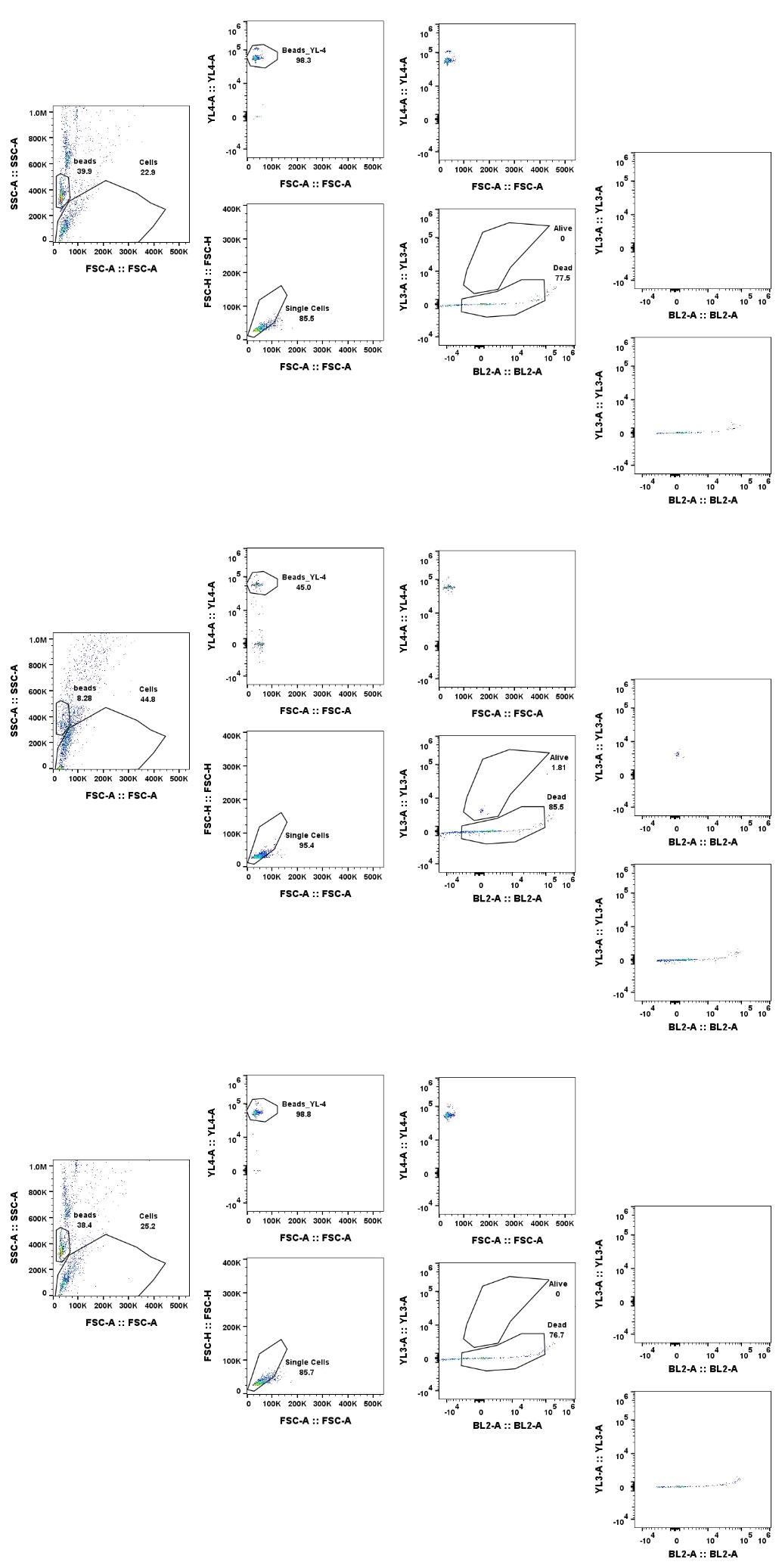


*Chroococcidiopsis cubana* PCC 7433: Escaped bacteria


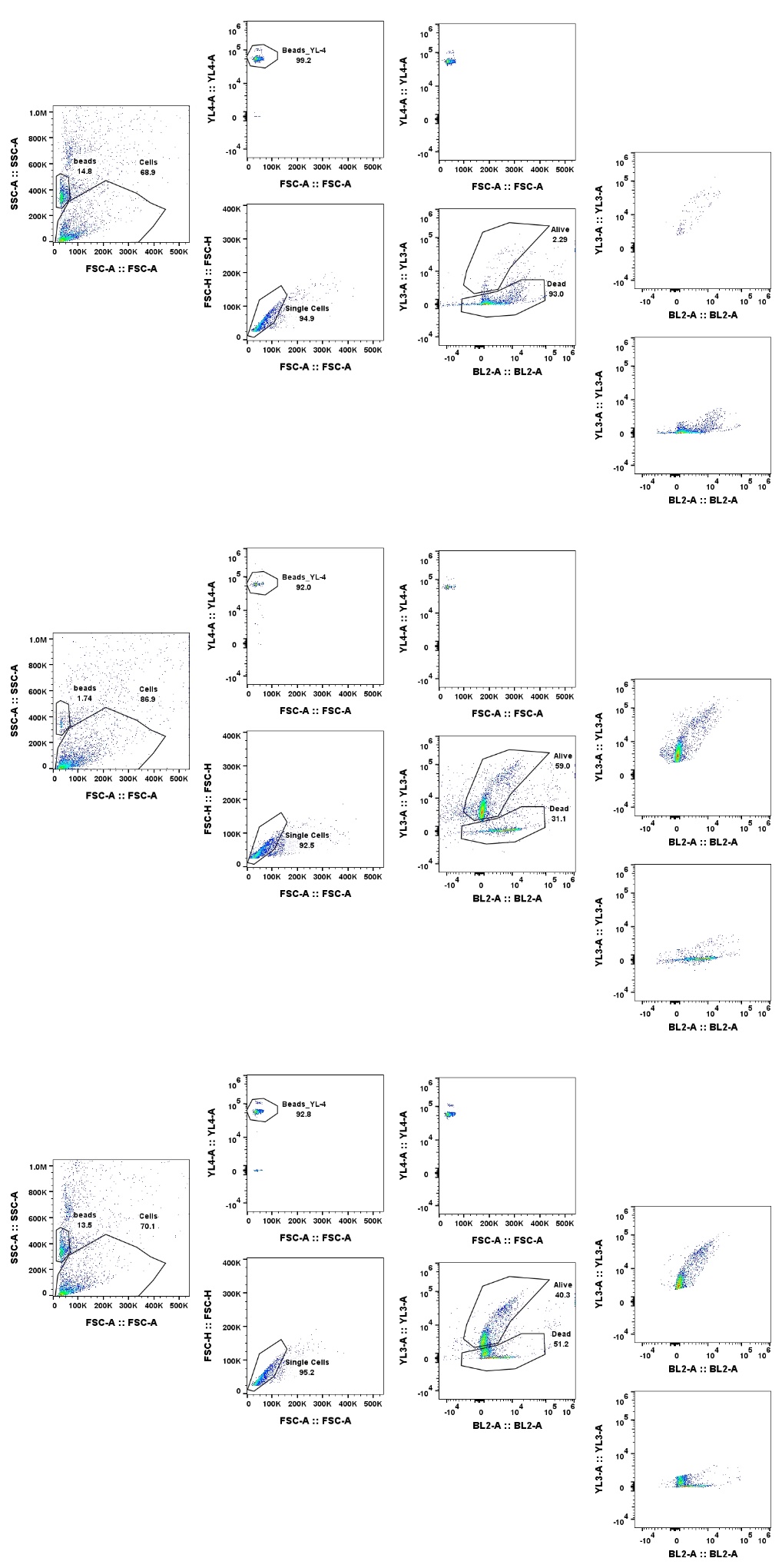


Long-term *Chroococcidiopsis* 7433 set-up: Live samples
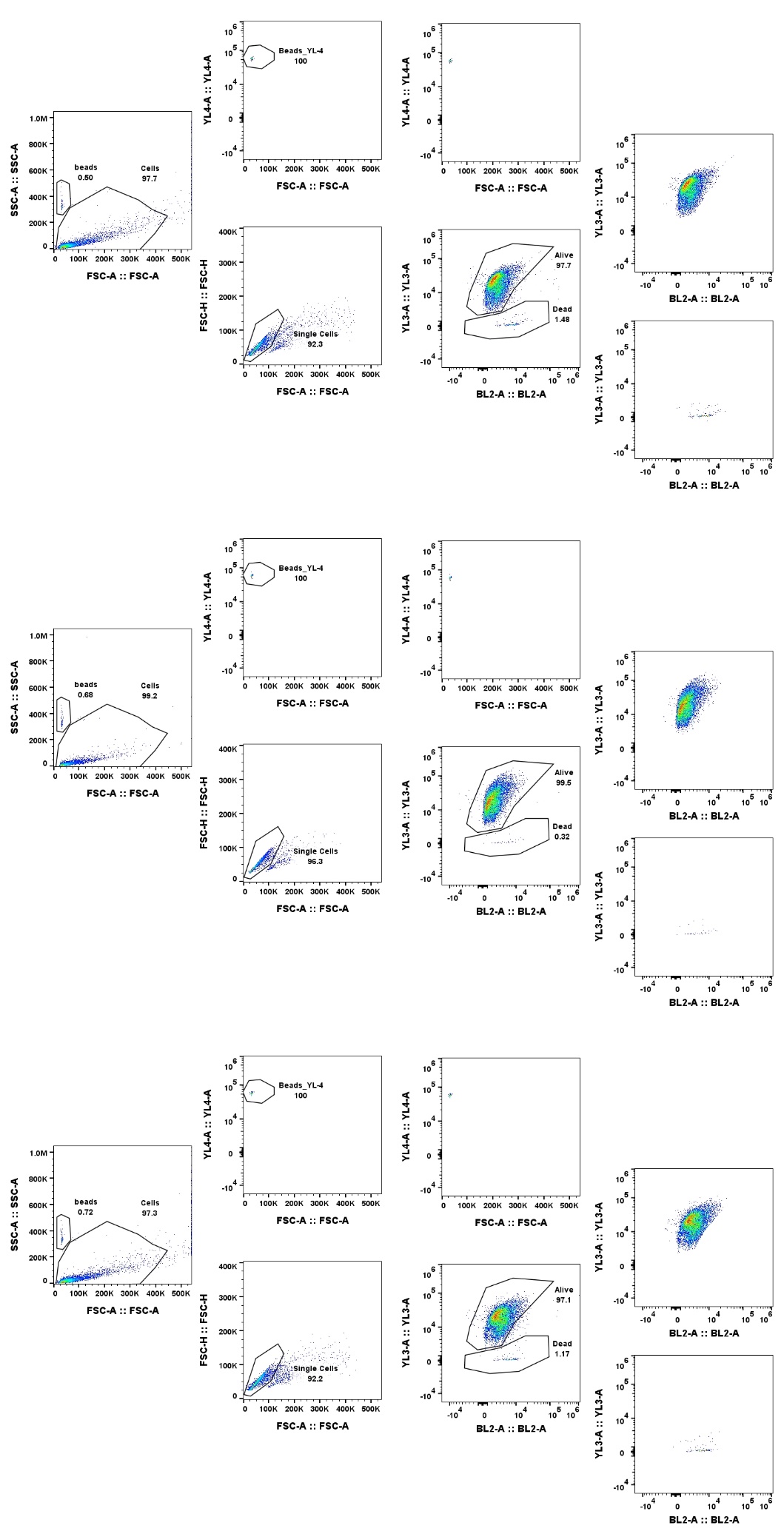


Long-term *Chroococcidiopsis* 7433 set-up: Killed samples


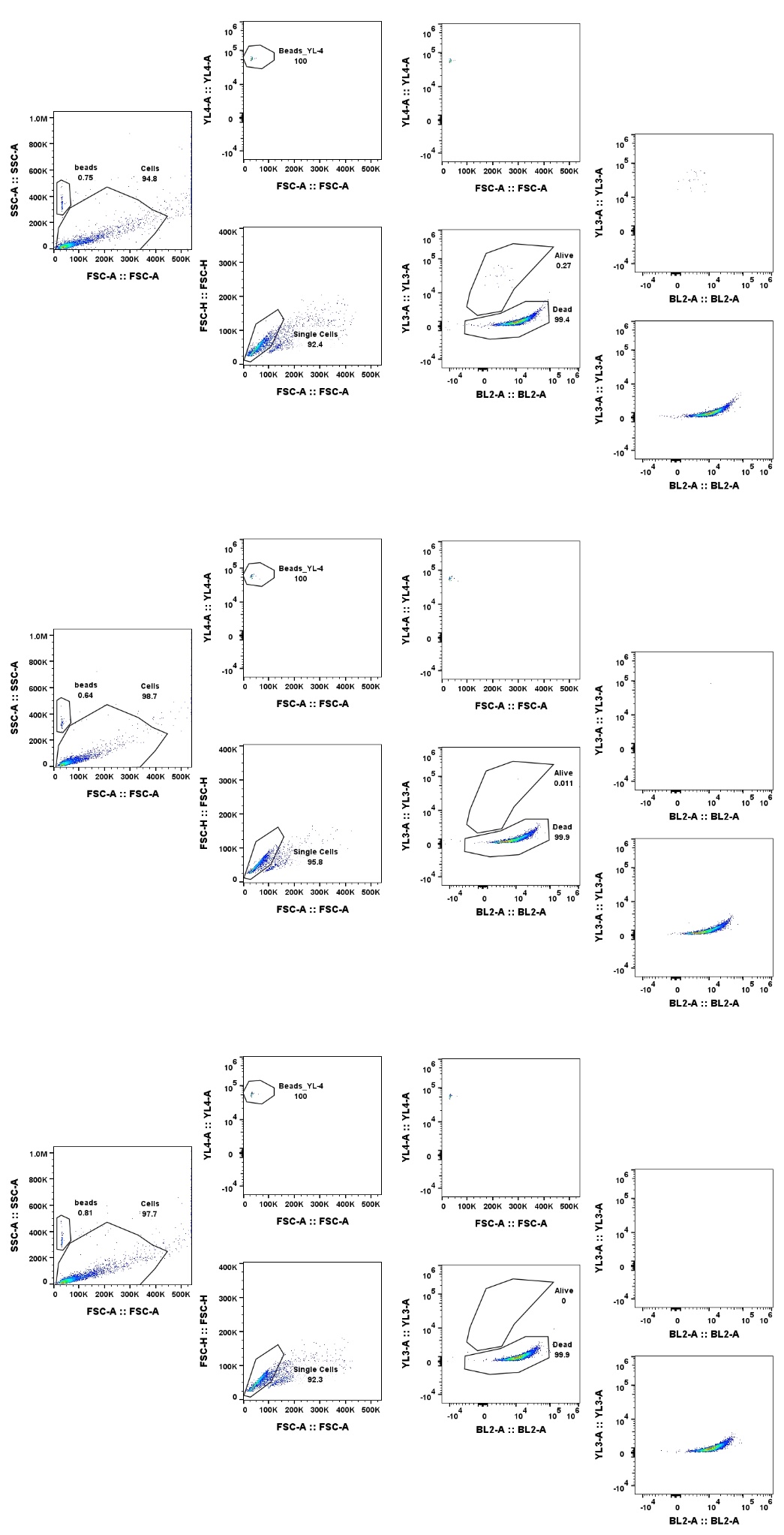


Long-term *Chroococcidiopsis* 7433 experiment: Escaped bacteria - First medium change D12


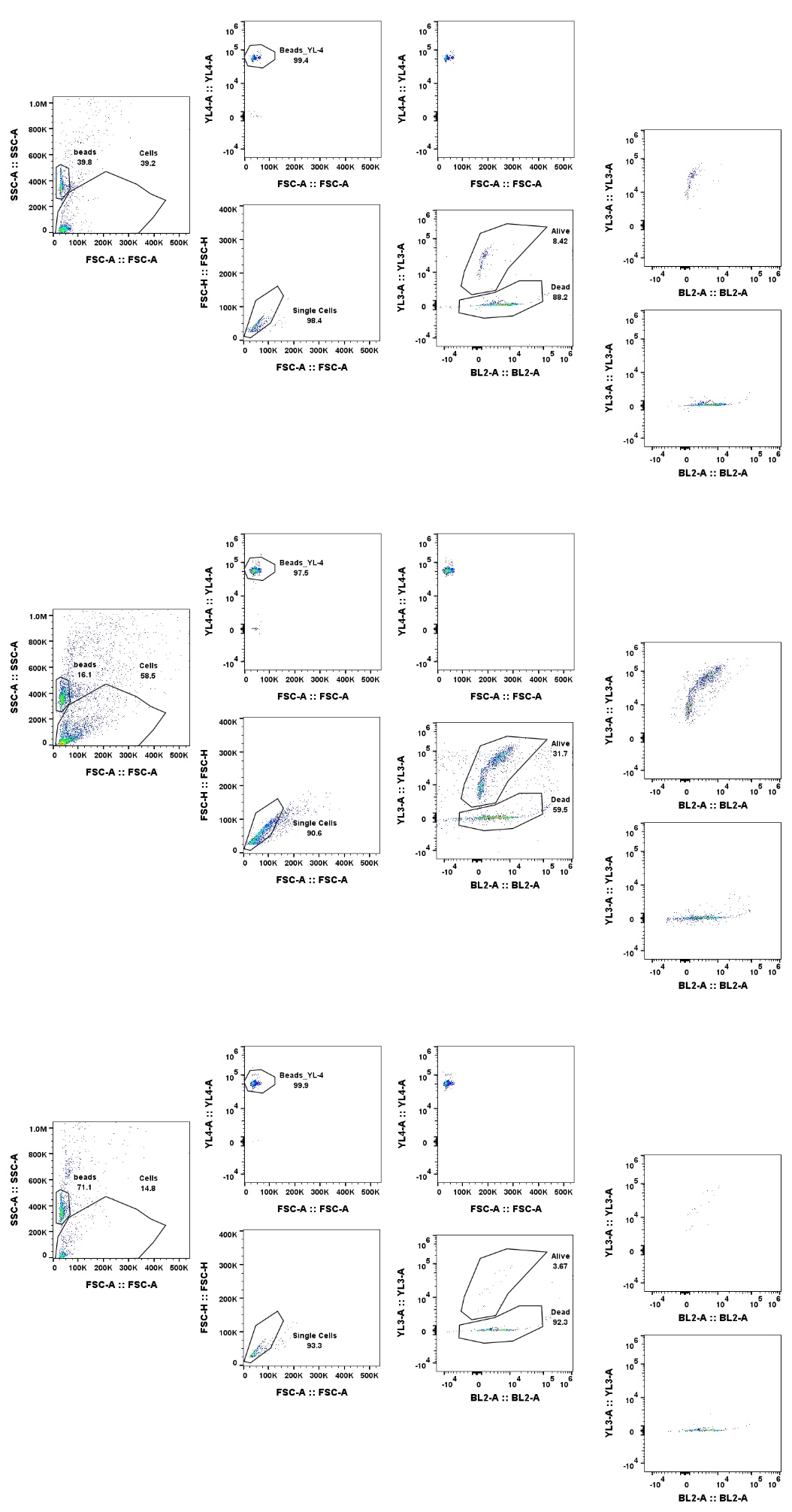


Long-term *Chroococcidiopsis* 7433 experiment: Escaped bacteria -Final medium change D27
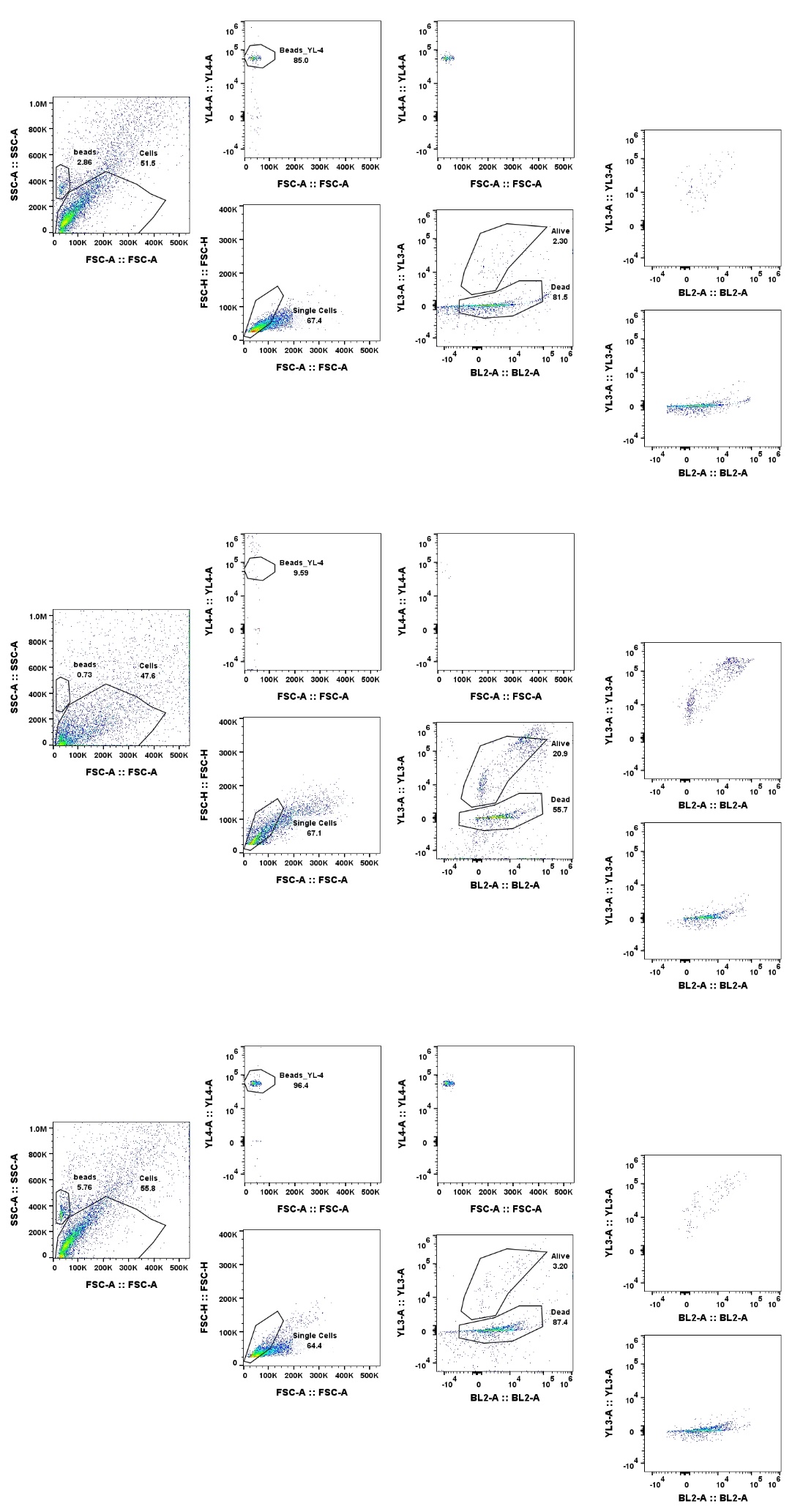


**References**

[1] Thermo Fisher Scientific. CountBright Absolute Counting Beads, https://www.thermofisher.com/order/catalog/product/C36950 (2005, accessed 27 January 2023).

[2] Thermo Fisher Scientific. CountBright^TM^ and CountBright^TM^ Plus Absolute Counting Beads, https://www.thermofisher.com/order/catalog/product/C36950 (2019, accessed 27 January 2023).
